# Limited role of mast cells during infection with the parasitic nematode *Litomosoides sigmodontis*

**DOI:** 10.1371/journal.pntd.0008534

**Published:** 2020-07-31

**Authors:** Lara Christine Linnemann, Martina Reitz, Thorsten B. Feyerabend, Minka Breloer, Wiebke Hartmann

**Affiliations:** 1 Bernhard Nocht Institute for Tropical Medicine, Hamburg, Germany; 2 Division for Cellular Immunology, German Cancer Research Center, Heidelberg, Germany; 3 Department of Biology, University of Hamburg, Hamburg, Germany; University of Zurich, SWITZERLAND

## Abstract

Mast cells are innate effector cells that due to their localization in the tissue form the first line of defense against parasites. We have previously shown that specifically mucosal mast cells were essential for the termination of the intestinal *Strongyloides ratti* infection. Here, we analyze the impact of mast cells on the immune response and defense against the tissue-dwelling filarial nematode *Litomosoides sigmodontis* using mast cell-deficient *Cpa3*^*cre*^ mice. Despite an increase and an activation of mast cells at the site of infection in wildtype BALB/c mice the outcome of *L*. *sigmodontis* infection was not changed in mast cell-deficient BALB/c *Cpa3*^*cre*^ mice. In *Cpa3*^*cre*^ mice neither vascular permeability induced by blood-sucking mites nor the migration of L3 was altered compared to *Cpa3* wildtype littermates. Worm burden in the thoracic cavity was alike in the presence and absence of mast cells during the entire course of infection. Although microfilaremiae in the peripheral blood increased in mast cell-deficient mice at some time points, the infection was cleared with comparable kinetics in the presence and absence of mast cells. Moreover, mast cell deficiency had no impact on the cytokine and antibody response to *L*. *sigmodontis*. In summary, our findings suggest that mast cells are not mandatory for the initiation of an appropriate immune response and host defense during *L*. *sigmodontis* infection in mice.

## Author summary

Mast cells are innate cells that are equipped with biologically potent granule proteins. Due to their localization in many tissues they form the first line of defense against parasites such as helminths. In the current study we analyzed the impact of mast cell deficiency on the course of a tissue-dwelling helminth infection. Mice were infected with the filarial nematode *L*. *sigmodontis* in the presence and absence of mast cells. We show that mast cell numbers increase at the site of infection and that mast cells are activated. Despite the recruitment of mast cells in infected wildtype BALB/c mice, worm burden in the thoracic cavity and final eradication of microfilariae from the peripheral blood were alike in mast cell-deficient and wildtype mice. Mast cell deficiency had no impact on the anti-helminth immune response. In summary, our findings suggest that mast cells are not required for a protective immune response against *L*. *sigmodontis* infection in mice.

## Introduction

Filarial nematodes such as *Brugia malayi*, *Wuchereria bancrofti* and *Onchocerca volvulus* are the causative agents of lymphatic filariasis and onchocerciasis, also known as riverblindness. The nematodes are transmitted by blood-sucking insects and it’s estimated that more than 100 million people suffer from these debilitating diseases [1]. Infection of BALB/c mice with the rodent nematode *L*. *sigmodontis* is the only fully permissive mouse model for human filariasis [2]. During a blood meal by mites (*Ornithonyssus bacoti*) third stage larvae (L3) are transmitted. The larvae migrate via the lymphatic vessels to the thoracic cavity, and develop into adult worms within 30 days. In susceptible BALB/c mice, male and female adults mate and the females release their offspring, the microfilariae (MF) or first stage larvae, into the circulation by day 55 post infection (p.i.). BALB/c mice remain infected for up to 200 days and thus represent an excellent model for chronic filarial infections [3].

Mast cells are innate effector cells that are located at the interface between the organs and the environment such as skin and gut [4]. Due to their localization mast cells are the first line of defense against invading pathogens. Mast cells are considered to be important sentinels and effector cells during infection with helminths [5]. Intestinal nematode infections have been mainly studied using *Kit*^*W*^*/Kit*^*W-v*^ and *Kit*^*W-sh*^ mice. However, since Kit-dependent ablation of mast cells leads to additional mast cell-independent immune deficiencies such as basocytopenia, neutropenia, anemia, impaired lymphocyte development and a loss of melanocytes, re-evaluation of mast cell functions is required [6]. Using a novel Kit-independent mast cell-deficient mouse model we have demonstrated that mucosal mast cells are non-redundant terminal effector cells during infection with the intestinal helminth parasite *Strongyloides ratti* [7]. The impact of the absence of mast cells on the immune response to tissue-dwelling filarial nematodes, however, has not been analyzed so far. A former study indicated a role of mast cells in the early phase of infection with *L*. *sigmodontis* [8]. Degranulation of mast cells and migration of larvae to the thoracic cavity are increased in CCL17-deficient mice. The phenotype in mice lacking this chemokine is reversed by chemical inhibition of mast cell degranulation.

Here, we investigate the role of mast cells during infection with the rodent filariae *L*. *sigmodontis* directly in a Kit-independent mouse model for mast cell deficiency. In *Cpa3*^*cre*^ mice, the heterozygous expression of the Cre recombinase under the control of the Carboxypeptidase A3 (Cpa3) promoter results in the depletion of mast cells and reduced numbers of basophils [9]. Despite the reduction of basophils, *Cpa3*^*cre*^ mice have an otherwise normal immune system and do not suffer from the severe side effects that had been described for the Kit-mutant mouse models [10, 11].

We show that although mast cells are recruited to the thoracic cavity and degranulate during infection with *L*. *sigmodontis* in BALB/c mice, their absence did not change infection outcome. We recorded no differences in the worm burden regarding fourth stage larvae (L4), adults as well as MF in mast cell-deficient *Cpa3*^*cre*^ in comparison to their mast cell-competent littermates. In line with this, the humoral and cellular immune response was similar in the presence and absence of mast cells. In summary our results suggest that mast cells are dispensable effector cells during infection with the tissue dwelling helminth parasite *L*. *sigmodontis*.

## Materials and methods

### Ethics and mice

Animal experimentation was conducted at the animal facility of the Bernhard Nocht Institute for Tropical Medicine in agreement with the German animal protection law under the supervision of a veterinarian. The experimental protocols have been reviewed and approved by the responsible federal health Authorities of the State of Hamburg, Germany, the "Behörde für Gesundheit und Verbraucherschutz" permission number 125/14.

BALB/c mice were obtained from Charles River (Sulzfeld, Germany). *Cpa3*^*cre*^ *mice* [9] on the BALB/c background have been described previously. The BALB/c *Cpa3*^*cre*^ mice were bred heterozygously (*Cpa3*^*cre/+*^ termed *Cpa3*^*cre*^ hereafter) in the animal facility of the Bernhard Nocht Institute for Tropical Medicine. To verify the genotype of the mice, DNA was extracted from earhole biopsies. *Cpa3*^*cre/wt*^ mice were genotyped by PCR using a combination of three oligonucleotides (common 5’: GGA CTG TTC ATC CCC AGG AAC C; 3’-WT: CTG GCG TGC TTT TCA TTC TGG;133’-KI: GTC CGG ACA CGC TGA ACT TG), yielding a 320 bp (Cpa3^+^) and 450 bp (Cpa3^cre^) product for heterozygous *Cpa3*^*cre*^ mice. Cycling conditions were as followed: 15 min polymerase activation at 95°C, 40 cycles at 95°C for 30 sec, 57°C for 30 sec and 72°C for 40 sec. Representative PCR products are shown in S1 Fig. Male and female wildtype (wt) littermates (termed *Cpa3*^*wt*^*)* were matched for gender and age and co-housed with the mast cell-deficient mice. All mice were kept in individually ventilated cages under specific pathogen-free conditions and were used at 7 to 10 weeks of age.

### Infection with *L*. *sigmodontis* and quantification of parasite burden

The life cycle of *L*. *sigmodontis* was maintained in cotton rats (*Sigmodon hispidus*), the natural reservoir of the nematode. Mites (*Ornithonyssus bacoti*), the intermediate hosts, were fed on infected cotton rats. 14 days after this blood meal mice were infected naturally by exposure to infected mites. Thereby mice belonging to different experimental groups were mixed and placed in the same tank to prevent a bias due to a different frequency or batches of mites. At day 10, 40 and 75 p.i. *Cpa3*^*cre*^ and their *Cpa3*^*wt*^ littermates were sacrificed. The number of worms was counted after flushing the thoracic cavity with 5–10 ml cold PBS. For analysis of day 10 fourth stage larvae, cells and worms were centrifuged and the pellet containing cells and larvae was resuspended in 1 ml PBS. Number of L4 was enumerated under the microscope in 96-well plates. For day 40 and 75 analysis, worms were counted in a petri dish. To detect MF in the circulation, blood of infected mice was collected in EDTA tubes. 20 μL of blood was added to 100 μL of ddH_2_O in order to lyse erythrocytes. The samples were centrifuged at 10,000 × g for 5 min. After discarding the supernatant, the pellet was resolved in 20 μL of Gentian violet and total number of MF in 20 μL blood was counted.

### Flow cytometry

Cells were stained with Live/Dead Fixable Blue Dead Cell Stain Kit (Life Technologies) or Zombie UV™ Fixable Viability Kit (Biolegend) according to the manufacturers’ instructions. For surface staining, cells were stained for 30 min on ice with FITC-labeled antibodies against CD4 (clone: RM4-5), CD8 (clone: 53–6.7) and CD19 cells (clone: 1D3), PerCP Cy5.5-labeled anti-mouse CD11b (clone: M1/70), PE-labeled anti-mouse IgE (clone: RME-1), Brilliant Violet 421-labeled anti-mouse CD117 (c-Kit; clone: 2B8). Ab were purchased from BioLegend or ThermoFisher Scientific. Samples were analyzed on a LSRII Flow Cytometer (Becton Dickinson) using FlowJo software (TreeStar).

### Quantification of the vascular permeability

For the *in vivo* degranulation assay 200 μL 1% Evans blue was injected i.v. into recipient mice (*Cpa3*^*cre/wt*^). Approximately 10–15 mites, that were fed on *L*. *sigmodontis*-infected or naïve cotton rats 14 days earlier, were collected into an Eppendorf tube. Infected and mock-infected mites were allowed to have a blood meal on the ear of anesthetized mice. After 5 min the tube and remaining mites were removed. As control an Eppendorf tube without mites was administered to the ears of control mice in parallel. 20 min later mice were killed by cervical dislocation. Ears were collected and dried overnight at 50°C. Evans blue was extracted by 24 h incubation of the chopped ears in 200 μL Formamid at 50°C. After centrifugation, the supernatant was transferred into a 96 well plate and the OD_630nm_ was measured.

### Quantification of the humoral immune response

For analysis of serum antibodies, blood was collected from mice by submandibular bleeding of the facial vein at day 40 and day 75 p.i. and allowed to coagulate for 1 h at room temperature. Serum was collected after centrifugation at 10,000 × g for 10 min and stored at −20°C for further analysis.

ELISA plates were coated overnight with 4 μg/mL *L*. *sigmodontis* antigen (LsAg) in PBS. Plates were washed, blocked by incubation with PBS 1% BSA for 2 h and incubated for 2 h with serum. After washing plates were incubated for 1 h with horseradish peroxidase-labeled anti-mouse IgG1, IgG2a and IgG2b (all Invitrogen), and IgE (BD Bioscience). Plates were washed and developed by incubation with 100 μL tetramethylbenzidine (0.1 mg/mL), 0.003% H_2_O_2_ in 100 mM NaH_2_PO_4_ pH 5.5 for 2.5 min. Reaction was stopped by addition of 50 μL 1 M H_2_SO_4_, and OD_450nm_ was measured. The more abundant isotype IgG1 was calculated by defining the highest serum dilution in a serial dilution (1:1000 to 1:128,000) resulting in an OD_450nm_ above the doubled background. For detection of LsAg-specific IgE, IgG2a and IgG2b a fixed serum dilution was used (1:100 for IgG2a and IgE and 1:1000 for IgG2b) and the background OD_450nm_ of the diluent (0.1% BSA in PBS) was subtracted.

### Quantification of the cytokine production and mast cell activation

For analysis of cellular responses mice were sacrificed at the indicated days p.i. and 5 × 10^5^ splenocytes were cultured in quadruplicates in 96-well round-bottom plates in RPMI 1640 medium supplemented with 10% FCS, 20 mM HEPES, L-glutamine (2 mM), and gentamicin (50 μg/mL) at 37°C and 5% CO_2_. The cells were stimulated for 72 h with medium, 12.5 μg/mL LsAg or 1 μg/mL anti-CD3. Cytokines in the culture supernatants were measured as described [12]. For analysis of mast cell degranulation, blood was collected from infected mice at the indicated time points and allowed to coagulate for 1 h at room temperature. Serum was harvested after centrifugation at 10,000 × g for 10 min. Mouse mast cell protease-1 (mMCPT-1) concentration was detected using the MMCP-I ELISA Ready-SET-Go kit (Invitrogen) according to the manufacturer’s recommendations.

### Statistical analysis

Samples were tested for Gaussian distribution and student’s t test (unpaired) or Mann-Whitney test were performed to compare 2 groups. One-way ANOVA with Bonferroni post test or Kruskal-Wallis Test with Dunn`s multiple comparison test was performed to compare more than 2 groups. A two-way ANOVA with Bonferroni posttest was conducted to compare two groups over time. Prism software Version 8 was used for statistical analysis (GraphPad Software). P-values ≤ 0.05 were considered statistically significant.

## Results

### Blood-sucking mites increase the vascular permeability independent of infective larvae

In the current study we used mast cell-deficient *Cpa3*^*cre*^ [9] mice, to analyze the function of mast cells during infection with *L*. *sigmodontis*.

**Fig 1:**
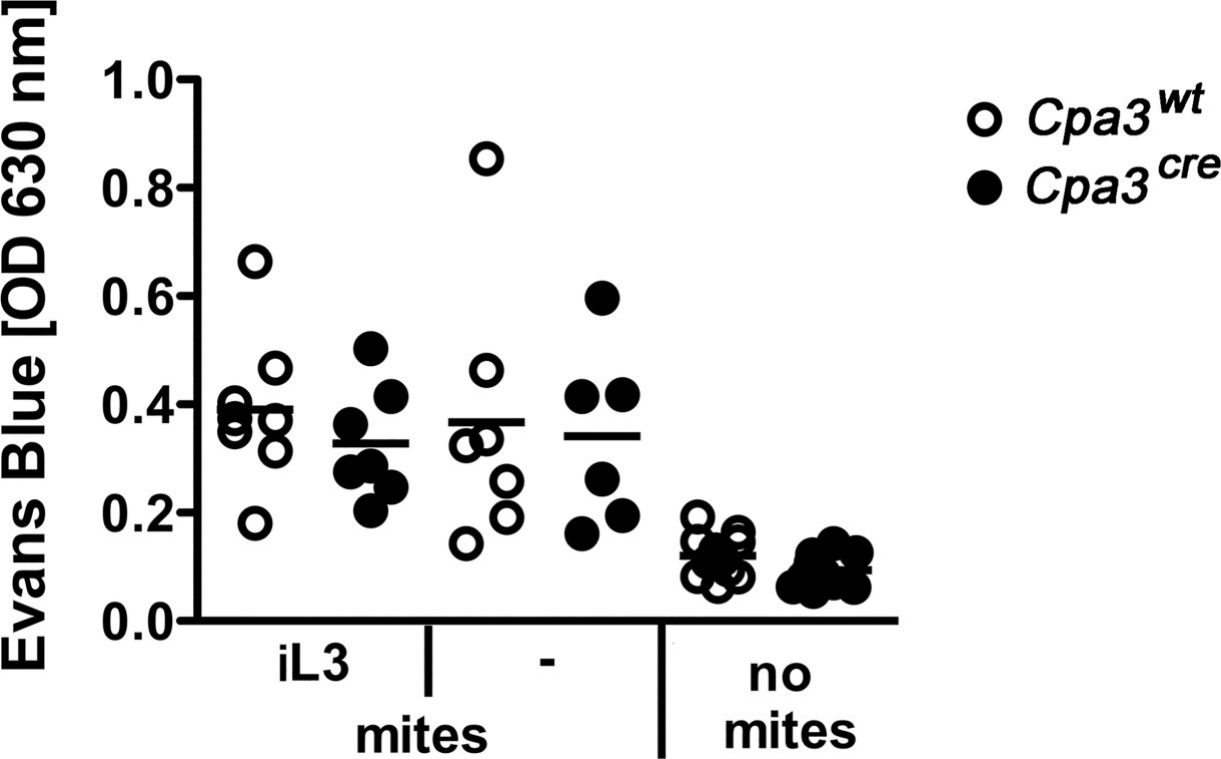
Mast cells have no impact on the vascular permeability induced by blood-sucking mites. Evans Blue was injected into the tail vein of mast cell-deficient *Cpa3*^*cre*^ and their competent littermates *Cpa3*^*wt*^ mice prior to a blood meal by mites. The blood-sucking mites were either infected (L3) or mock-infected (-) 14 days earlier. Evans Blue was extracted from the ears, as control the contralateral ear of the mice was taken (no mites). Each dot represents values derived from a single mouse (L3-infected mites: n = 8 *Cpa3*^*wt*^ *and n = 7 Cpa3*^*cre*^ mice; mock-infected mites: n = 7 *Cpa3*^*wt*^ *and n = 6 Cpa3*^*cre*^ mice; no mites: n = 13 *Cpa3*^*wt*^ *and Cpa3*^*cre*^ mice).

During a natural infection with *L*. *sigmodontis* blood-sucking mites transmit L3 into the skin of recipient mice. To analyze whether this blood meal triggers a local activation of mast cells, we compared the vascular permeability in mast cell-deficient mice and their cell-competent littermates. Blood-sucking mites increased the vascular permeability indicated by an increased extravasation of Evans blue into the tissue in BALB/c *Cpa3*^*cre*^ mice and their respective wt littermates (Fig 1). Thereby exposure to mock-infected mites and L3-infected mites increased the vascular permeability at the site of the blood meal in mast cell-competent and mast cell-deficient mice to the same extent. The observed increase in vascular permeability was specifically induced by the blood meal of mites as no change in the barrier function of the tissue was measured in the contralateral, non-exposed ear (Fig 1, no mites). Thus, feeding mites locally enhance the vascular permeability during their blood meal independent of the presence of L3 or mast cells.

### Mast cells increase at the site of infection and are activated

After being transmitted during the blood meal, L3 migrate within 3–5 days to the thoracic cavity. We infected BALB/c mice with *L*. *sigmodontis* and performed flow cytometric analyses in the early infection phase at day 8 p.i. (L3/L4), at day 30 p.i. (immature adult worms), at day 60 p.i. (onset of patency) and at day 90 p.i. (chronic phase), to analyze whether mast cells are recruited to the site of infection. Mast cells are defined as lineage and CD11b negative cells. The lineage cocktail included antibodies against CD4, CD8 and CD19. Mast cells are positive for c-Kit (CD117) and IgE, the gating strategy is shown in S2 Fig.

**Fig 2:**
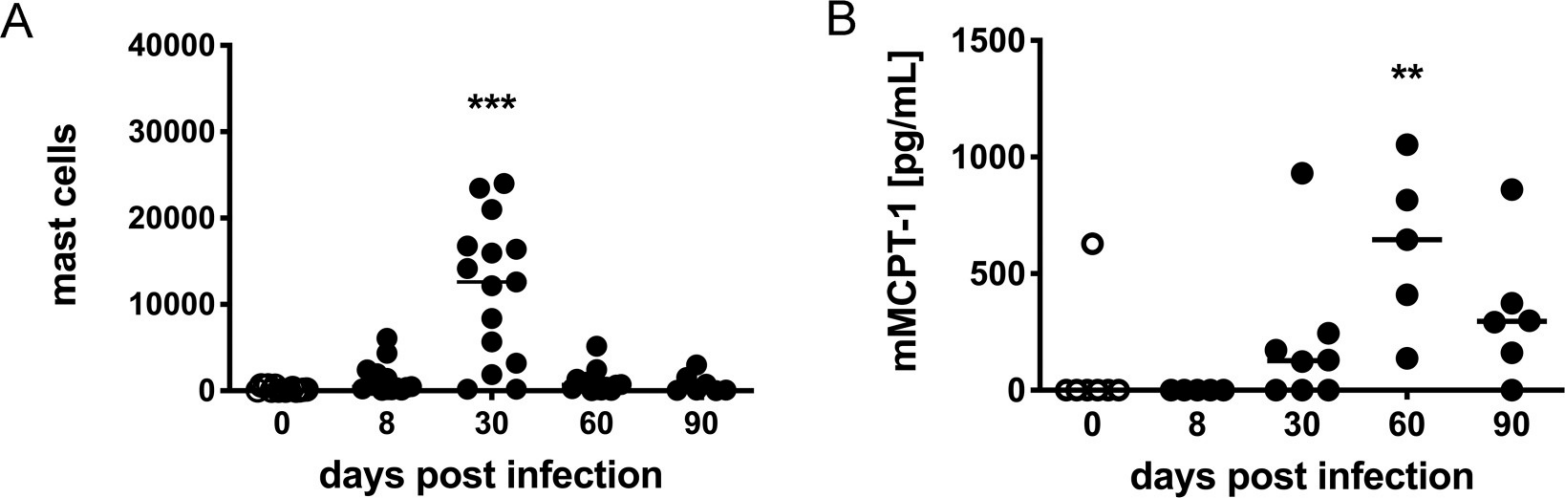
Mast cells are recruited and activated during the course of infection. BALB/c mice were infected with *L*. *sigmodontis* and mice were sacrificed at different times post infection. A: Number of mast cells in the thoracic cavity. B: mMCPT-1 was measured in the blood of infected mice in comparison to noninfected (day 0 p.i.) mice. Each dot represents a value from a single mouse (A: day 0 n = 15, day 8 n = 13, day 30 n = 15, day 60 n = 13, day 90 n = 7 mice; B: day 0 n = 7, day 8 n = 5, day 30 n = 8, day 60 n = 5, day 90 n = 6 mice). Asterisks indicate significant differences of a particular day p.i. compared to noninfected (day 0 p.i.) mice. ** p ≤ 0.01; *** p ≤ 0.001.

The number of mast cells drastically increased at day 30 p.i., while at day 8, 60 and 90 p.i. mast cell numbers were only increased by trend (Fig 2A) in BALB/c mice. To analyze the activation status of mast cells during *L*. *sigmodontis* infection, we recorded the concentration of mMCPT-1, a protease that is released by degranulating mucosal mast cells [13]. Elevated mMCPT-1 levels in the serum were detectable with a delayed kinetic by day 60 p.i. (Fig 2B) indicating either the systemic accumulation of mMCPT-1 after local degranulation of mast cells in the thoracic cavity and/or additional activation of mast cells at other sites such as the lung or the peritoneal cavity.

### Mast cells are dispensable for the control of *L*. *sigmodontis* infection

**Fig 3:**
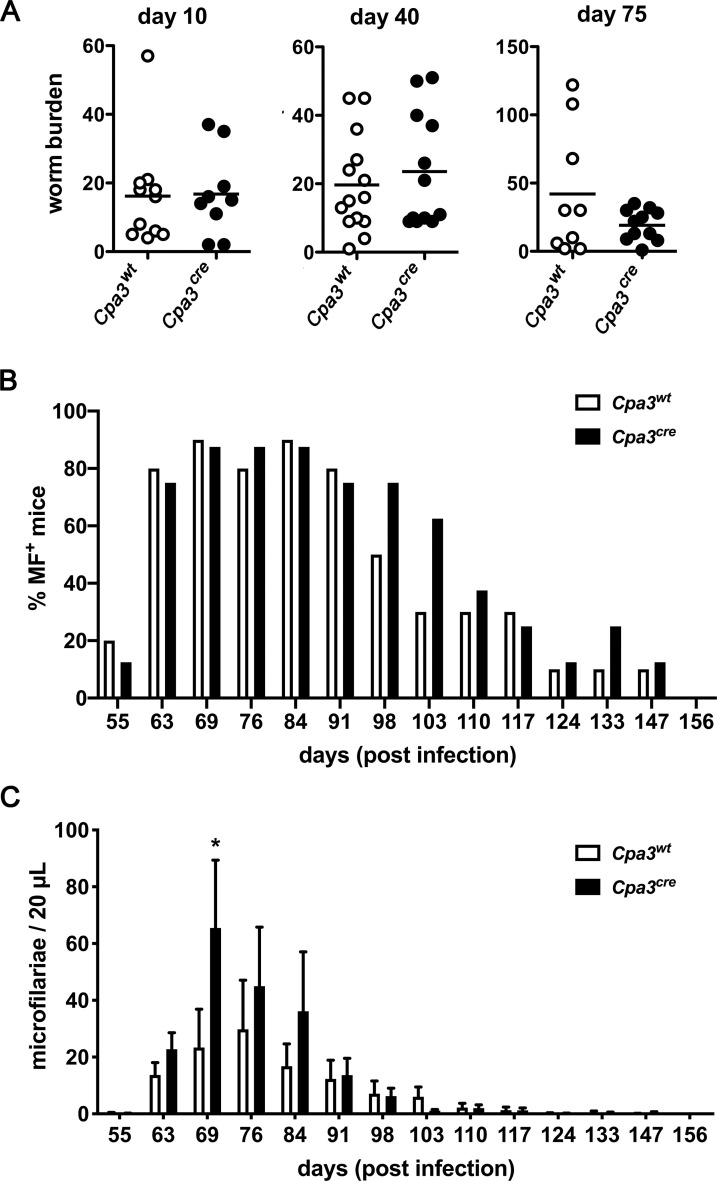
The course of infection is not altered in the absence of mast cells. Mast cell-deficient *Cpa3*^*cre*^ and their cell-competent littermates *Cpa3*^*wt*^ mice were infected with *L*. *sigmodontis*. A) The number of worms was counted at the indicated time points. Each dot represents the worm burden from one single mouse (day 10: n = 11 *Cpa3*^*wt*^ *and n = 9 Cpa3*^*cre*^; day 40: n = 14 *Cpa3*^*wt*^ *and n = 12 Cpa3*^*cre*^; day 75: n = 9 *Cpa3*^*wt*^ *and n = 12 Cpa3*^*cre*^ *mice)*. Data show the mean and were pooled from 2–3 experiments. Microfilariae were counted at the indicated time points. B) The y-axis shows the frequency of microfilaremic mice over time. C) The number of MF in 20 μL peripheral blood. Data show the mean and the SEM and are combined from two independent experiments with n = 10 *Cpa3*^*wt*^ and n = 8 *Cpa3*^*cre*^ mice. Microfilaremiae was analyzed with a two-way ANOVA with Bonferroni post test.

To elucidate the role of mast cells in the control of *L*. *sigmodontis* we naturally infected *Cpa3*^*cre*^ mice, which lack mucosal and connective tissue mast cells, as well as their mast cell-competent *Cpa3*^*wt*^ littermates with *L*. *sigmodontis*. After exposure to infected mites we recorded the parasite burden at several time points. Thereby we analyzed day 10 p.i. to count *L*. *sigmodontis* L3 at the transition to L4, day 40 to count mature adults and day 75 to count the mature reproducing adults in the thoracic cavity. The number of worms in the thoracic cavity was similar during all stages of infection in mast cell-deficient mice compared to their mast cell-competent littermates (Fig 3A). Next, we monitored the release of MF into the circulation. In total, 9 out of 10 *Cpa3*^*wt*^ and all 8 *Cpa3*^*cre*^ mice had detectable numbers of MF in the blood. The distribution of MF^+^ mice was similar in both mouse strains over time (Fig 3B). *Cpa3*^*cre*^ mice had significantly more MF at day 69 p.i., and displayed slightly elevated MF numbers in the blood at days 76 and 84 p.i. However, MF numbers at later time points were unchanged and final clearance of MF from the circulation occurred with similar kinetics in *Cpa3*^*cre*^ and *Cpa3*^*wt*^ mice (Fig 3C).

Taken together, although mast cells were recruited to the thoracic cavity (Fig 2A), and were activated (Fig 2B) during infection of wt BALB/c mice with *L*. *sigmodontis*, the genetic ablation of mast cells in *Cpa3*^*cre*^ mice resulted in a transient elevation of microfilaraemia but did not interfere with successful and timely termination of the parasite infection.

### *L*. *sigmodontis-*specific immune response is not altered by the absence of mast cells during primary infection

**Fig 4:**
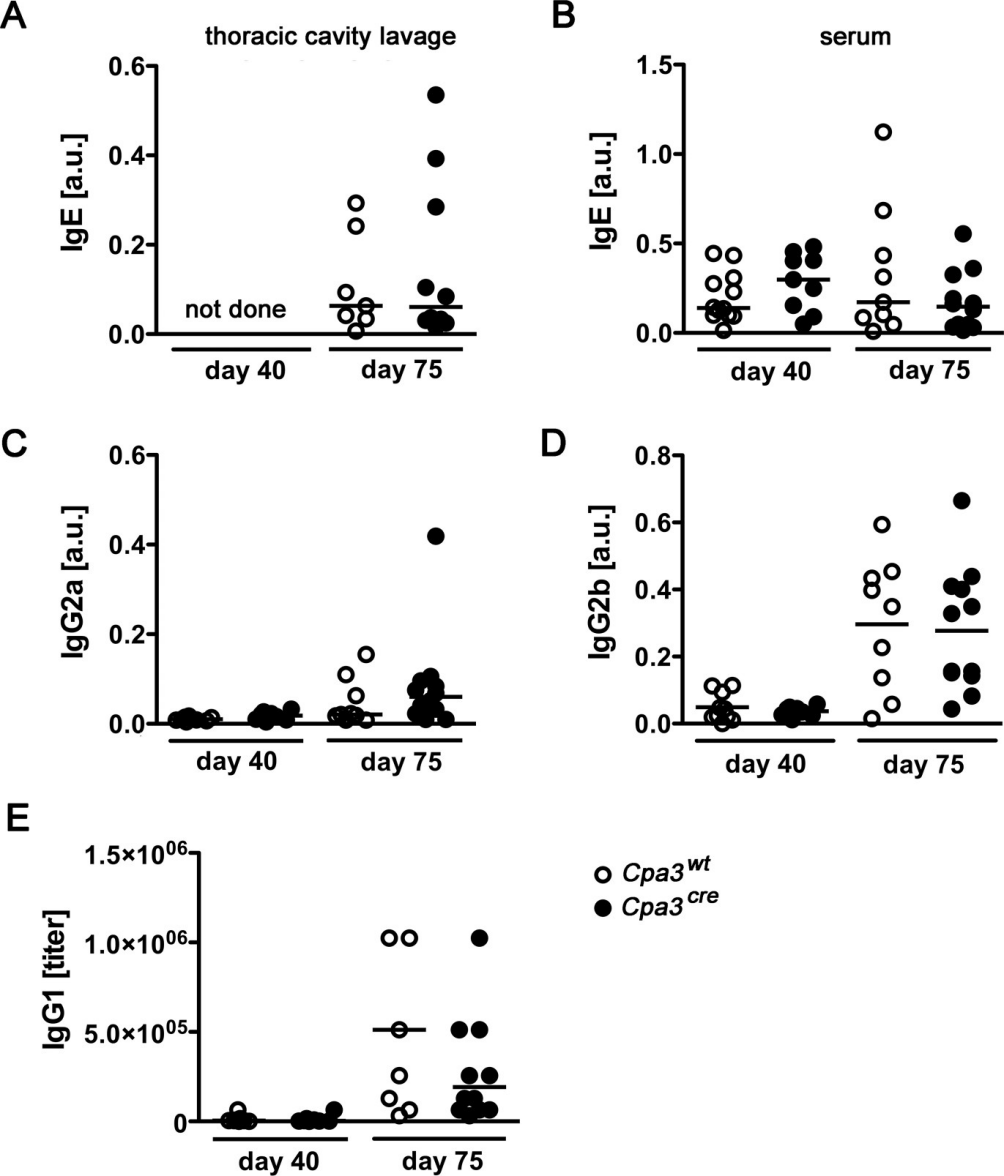
*L*. *sigmodontis*-specific Ig response is not altered by the absence of mast cells. Mast cell deficient *Cpa3*^*cre*^ and their cell-competent littermates *Cpa3*^*wt*^ mice were infected with *L*. *sigmodontis* for 40 and 75 days. A) LsAg-specific IgE was analyzed by ELISA in the thoracic cavity lavage (A) and the serum (B) of infected mice. LsAg-specific IgG2a (C), IgG2b (D), and IgG1 (E) was measured in the serum of infected *Cpa3*^*cre*^ and *Cpa3*^*wt*^ mice. Data show the mean and are combined from two independent experiments. Each dot represents the Ig response from a single mouse (A: day 75: n = 7 *Cpa3*^*wt*^ and n = 10 *Cpa3*^*cre*^*; B* day 40: n = 12 *Cpa3*^*wt*^ *and n = 9 Cpa3*^*cre*^; day 75: n = 9 *Cpa3*^*wt*^ *and n = 12 Cpa3*^*cre*^; C-E: day 40: n = 10 *Cpa3*^*wt*^ *and n = 9 Cpa3*^*cre*^; day 75: n = 9 *Cpa3*^*wt*^ *and n = 12 Cpa3*^*cre*^ mice*)*. The less abundant isotypes IgE and IgG2 are expressed as a.u., IgG1 data is depicted as titer. Data were analyzed by unpaired student t test or Mann-Whitney U test.

Since mast cells have been proposed to orchestrate type 2 immune responses during helminth infection [14, 15] we determined the *L*. *sigmodontis*-specific cellular and humoral immune responses. *Cpa3*^*cre*^ and *Cpa3*^*wt*^ mice were infected for 40 or 75 days and the *L*. *sigmodontis*-specific Ig responses directed against adult worm extract were analyzed. LsAg-specific IgE in the thoracic cavity lavage and the serum was similar in *L*. *sigmodontis*-infected *Cpa3*^*cre*^ and *Cpa3*^*wt*^ mice at day 40 and day 75 p.i. (Fig 4A and 4B). Th1-associated LsAg-specific IgG2 was low but similar in *L*. *sigmodontis*-infected *Cpa3*^*cre*^ and *Cpa3*^*wt*^ mice: LsAg-specific IgG2a was barely detectable (Fig 4C), while LsAg-specific IgG2b increased slightly at day 75 p.i. (Fig 4D). LsAg-specific IgG1 was the most prominent isotype that increased at day 75 p.i. in mast cell-deficient and mast cell–competent mice to the same extent (Fig 4E). Thus, altogether the type 2 profile of the LsAg-specific Ig response was not altered by mast cell deficiency.

**Fig 5:**
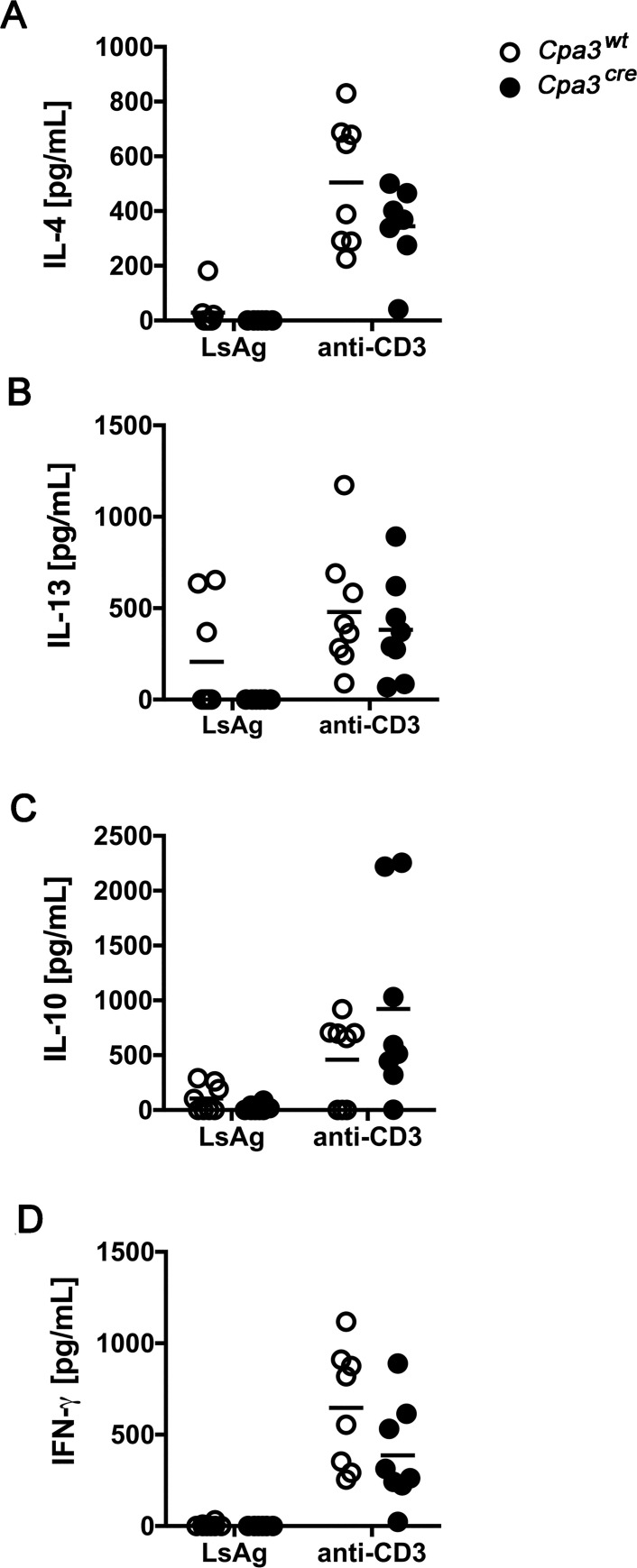
The cytokine response is not altered by the absence of mast cells. Mast cell deficient *Cpa3*^*cre*^ and their cell-competent littermates *Cpa3*^*wt*^ mice were infected with *L*. *sigmodontis* for 40 days. IL-4 (A), IL-13 (B), IL-10 (C) and IFN-γ (D) were measured by ELISA in the supernatant of spleen cells after stimulation with LsAg or anti-CD3. Each dot represents a value from a single mouse, the lines represent the mean. Data are combined from two independent experiments (n = 8 *Cpa3*^*wt*^ *and n = 8 Cpa3*^*cre*^ mice) and were analyzed by unpaired student t test.

We next compared the T cell cytokine response in mast cell-deficient and mast cell-competent littermates by measuring cytokines in the supernatant of spleen cells. Spleen cells were isolated from *L*. *sigmodontis*-infected mice at day 40 p.i. and stimulated *ex vivo*. *L*. *sigmodontis* antigen-specific and anti-CD3-induced cytokine production by spleen cells were unchanged in mast cell-deficient *Cpa3*^*cre*^ and mast cell-competent *Cpa3*^*wt*^ mice (Fig 5). Spleen cells from mast cell-deficient *Cpa3*^*cre*^ mice showed very low, but comparable *L*. *sigmodontis* antigen-specific release of the Th2-cytokines IL-4 (Fig 5A) and IL-13 (Fig 5B), of the regulatory cytokine IL-10 (Fig 5C), and the Th1-associated cytokine IFN-γ (Fig 5D) as their *Cpa3*^*wt*^ littermates. Polyclonal stimulation of T cells with anti-CD3 resulted in strong production of all cytokines measured. Thereby IL-4 and IFN-γ synthesis were slightly but statistically not significantly decreased in mast cell-deficient *Cpa3*^*cre*^ mice compared to *Cpa3*^*wt*^ mice (Fig 5A and 5D). The concentration of IL-10 and IL-13 was comparable in the supernatants from anti-CD3 stimulated spleen cells from *Cpa3*^*cre*^ and *Cpa3*^*wt*^ mice (Fig 5B and 5C).

In summary, mast cell deficiency did not dramatically alter the initiation of a mixed type 1 and type 2 immune response directed against *L*. *sigmodontis*.

## Discussion

Since mast cells contribute to immunity against intestinal helminth infections, we aimed to investigate their role during infection with the tissue dwelling filarial nematode *L*. *sigmodontis*. To this end, we performed natural infections via blood sucking mites that transmit *L*. *sigmodontis* L3 to the host. We have previously shown that basophils have no impact on the vascular permeability and thereby are not required during migration of L3 to the thoracic cavity [16]. Like basophils mast cells contain granule proteins that increase vascular permeability. We hypothesized that the absence of mast cells in fully susceptible BALB/c *Cpa3*^*cre*^ mice would result in decreased vascular permeability and an impaired migration of larvae to the thoracic cavity. Surprisingly, the increase of vascular permeability, induced by blood-sucking mites, was independent of both, mast cells and the presence of L3. Vasoactive substances in the saliva, that are released during blood-feeding of arthropod vectors, have been shown to increase vascular permeability in the host [17]. Studying natural infections we cannot formally rule out that isolated L3 have subtle effects on vascular permeability that are masked by the vasoactive substances present in the saliva of the arthropod vector [17]. Nevertheless, transferred to humans, our data suggest that the impact of saliva components would dominate any effect by L3 on vascular permeability. Additionally, mast cell deficiency did not affect the migratory capacity of L3 to the thoracic cavity since a similar number of L3 developed into L4 at day 10 p.i. in mast cell-deficient BALB/c *Cpa3*^*cre*^ and mast cell-competent BALB/c *Cpa3*^*wt*^ mice. A previous study indicated a role of mast cells in promoting L3 migration to the thoracic cavity in the absence of the chemokine CCL17 in C3H/HeN mice [8]. In CCL17-deficient mice increased mast cell numbers in the skin were associated with increased vascular permeability and elevated parasite loads at day 10 p.i. Thus, it was suggested that CCL17 would control mast cell degranulation and limit vascular permeability in the wildtype situation. Our results using BALB/c *Cpa3*^*cre*^ mice confirm a neglectable role of skin mast cells in the vascular permeability and consequently migration of larvae to the thoracic cavity in immunocompetent mice.

Once the larvae arrive in the thoracic cavity, they are continuously attacked by the immune system and are cleared within 200 days in the context of a mixed type 1 and 2 immune response [2]. In the wildtype situation, we observed a recruitment of mast cells in BALB/c mice to the site of infection, displaying maximal numbers at day 30 p.i. In addition, mast cells were activated during the course of infection indicated by release of mMCPT-1 in the serum of infected BALB/c mice. We have previously shown that IgE, that is known to activate mast cells by crosslinking of the high-affinity IgE receptor, is increased during *L*. *sigmodontis* infection as well [16]. Nevertheless, the absence of mast cells in *Cpa3*^*cre*^ mice did neither change the vascular permeability induced by blood-sucking mites nor the number of L4 or adult parasites in the thoracic cavity at any time point during *L*. *sigmodontis* infection. We recorded a transient increase of microfilariae at day 69 p.i. in mast cell-deficient *Cpa3*^*cre*^ mice compared to wildtype mice. Regardless of the increased number, however, MF were cleared from the circulation with similar kinetics in mast cell-deficient and mast cell-competent mice. Despite the presence and activation of mast cells at the site of *L*. *sigmodontis* infection in wildtype BALB/c mice, our data using mast cell-deficient *Cpa3*^*cre*^ mice suggest that mast cells are dispensable for the outcome of the infection and the immune parameters analyzed in this study.

A recent study linked protection from anaphylaxis in *L*. *sigmodontis*-infected mice in an OVA model to decreased peritoneal mast cell numbers, less granularity and an overall less activated phenotype at day 112 p.i. [18]. The authors suggested that mast cells might become constantly activated during chronic infection with *L*. *sigmodontis*. Crosslinking of IgE by excretory-secretory products that are released by *L*. *sigmodontis* might thereby lead to some kind of anergic/exhausted mast cell phenotype. We observed mast cell expansion and activation during *L*. *sigmodontis* infection in the current study. Moreover, we have previously shown an increase of polyclonal IgE in the serum and an increase of *L*. *sigmodontis*-specific IgE locally in the thoracic cavity [16] until day 90 p.i. Thus, a repeated activation of mast cells during chronic *L*. *sigmodontis* infection is in line with this recent study [18] principally conceivable.

Mast cells have been shown to be directly toxic to nematodes by release of proteases [19]. In addition, they contribute to expulsion of intestinal helminth parasites by goblet cell hyperplasia, increased mucus production and smooth muscle cell contraction [5]. Therefore, mast cell-driven expulsion of nematode species that are located in the intestine but to a lesser extent to nematodes dwelling in the thoracic cavity is likely. The role of mast cells as effector cells during infection with intestinal helminth parasites is clearly established for *S*. *ratti* [7] and *S*. *venezuelensis* [20] using Kit-independent mouse models for mast cell deficiency. Studies using Kit-dependent mast cell-deficient mice further indicate a role of mast cells during infection with *H*. *polygyrus*, *Trichuris muris* [14] and *Hymenolepsis diminuta* infection [15]. In contrast, expulsion of *N*. *brasiliensis* did not require mast cells [21]. In addition, mast cells have been described to initiate early anti-*H*. *polygyrus* and *T. muris* immune responses [14]. Thereby in mast cell-deficient Kit^W^/Kit^W-v^ and Kit^W-sh^ Kit mice early production of alarmins triggered by degranulating mast cells and subsequent Th2 priming was compromised. By contrast, the initiation of the mixed Th1/2 immune response during *L*. *sigmodontis* infection was similar in the presence and absence of mast cells. In line with these findings, the immune response against the intestinal parasite *S*. *ratti* was unchanged in mast cell-deficient mice [7]. While our data argue against a central impact of mast cells in the initiation of anti-helminth immune responses, it should be noted that we cannot formally exclude compensatory mechanisms by other mast cell like cells that could mask a putative phenotype in mast cell-deficient *Cpa3*^*cre*^ mice.

However, using the same mouse model for mast cell deficiency we demonstrated a central role for mast cells as effector cells during infection with the intestinal nematode *S*. *ratti*. Hereby, mast cell-deficient *Cpa3*^*cre*^ mice were unable to expulse parasitic adults from the intestine and did not terminate an infection with *S*. *ratti* for more than 150 days while mast cell-competent *Cpa3*^*wt*^ littermates cleared the infection after a month [7]. A role for basophils as redundant effector cells seems unlikely since we have previously shown, that these cells are dispensable during infection with *L*. *sigmodontis* [16].

We haven’t analyzed the contribution of mast cells to lung pathology in the current study. During the course of infection L3 migrate via the lung to the thoracic cavity. This pulmonary phase is associated with lung inflammation and recruitment of neutrophils [22]. Since mast cells have been shown to interact with neutrophils and promote their extravasation in various settings [23], mast cells might be involved in lung damage in *L*. *sigmodontis*-infected mice. Additionally, the presence of MF in chronically *L*. *sigmodontis*-infected BALB/c mice was linked to increased mucus production and goblet cell hyperplasia in the bronchial epithelium [24]. Mast cells, thus, might play a role in lung pathology rather than immunity during *L*. *sigmodontis* infection.

In summary, combined data from others and us provide accumulating evidence that mast cells increase at the site of helminth infection and are activated by both, tissue-dwelling and intestinal parasites. However, despite their role in the initiation and execution of anti-helminth immune responses during a first infection with several intestinal helminth parasites, mast cells are not central during infection with the tissue-dwelling nematode *L*. *sigmodontis*.

## Supporting information

S1 Fig To verify the genotype of the mice, DNA was extracted from earhole biopsies. *Cpa3*^*cre/wt*^ mice were genotyped by PCR using a combination of three oligonucleotides (common 5’: GGA CTG TTC ATC CCC AGG AAC C; 3’-WT: CTG GCG TGC TTT TCA TTC TGG;133’-KI: GTC CGG ACA CGC TGA ACT TG), yielding 320 bp (Cpa3^+^) and 450 bp (Cpa3^cre^) products for heterozygous *Cpa3*^*cre*^ mice. Cycling conditions were as followed: 15 min polymerase activation at 95°C, 40 cycles at 95°C for 30 sec, 57°C for 30 sec and 72°C for 40 sec.
(PDF)

S2 Fig Gating strategy. Representative dot blots showing the gating strategy. Thoracic cavity cells were isolated from noninfected mice (A) and day 30 *L*. *sigmodontis* infected mice (B). Mast cells were defined as lineage (CD4, CD8 and CD19)^-^ CD11b^-^ leukocytes that are c-Kit (CD117)^+^ and IgE^+^. 1 x 10^6^ cells were gated for the analysis.
(PDF)
