# Supplementary material for: Limited role of mast cells during infection with the parasitic nematode Litomosoides sigmodontis
Source: PLoS Negl Trop Dis. 2020 Jul 31;14(7):e0008534. doi: 10.1371/journal.pntd.0008534 (PMC7423137; doi:10.1371/journal.pntd.0008534)
Supplement: S1 Fig — Cpa3cre/wt mice were genotyped by PCR using a combination of three oligonucleotides (common 5’: GGA CTG TTC ATC CCC AGG AAC C; 3’-WT: CTG GCG TGC TTT TCA TTC TGG;133’-KI: GTC CGG ACA CGC TGA ACT TG), yielding 320 bp (Cpa3+) and 450 bp (Cpa3cre) products for heterozygous Cpa3cre mice. Cycling conditions were as followed: 15 min polymerase activation at 95°C, 40 cycles at 95°C for 30 sec, 57°C for 30 sec and 72°C for 40 sec. (PDF) [file pntd.0008534.s001.pdf]

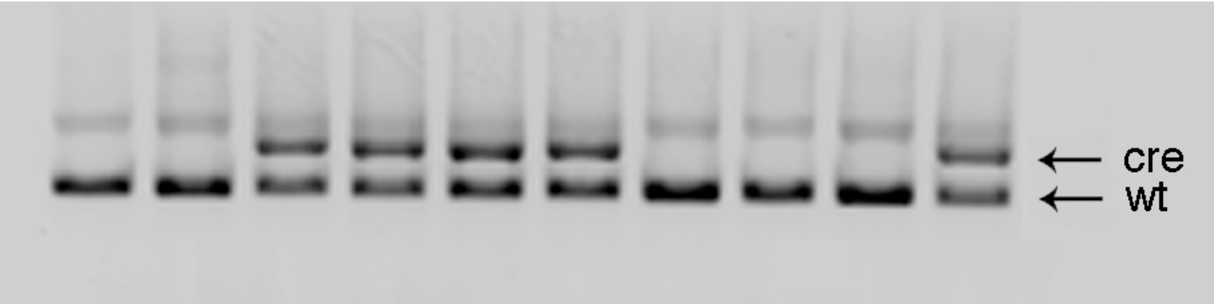

S1 Fig: PCR for genotyping.

To verify the genotype of the mice, DNA was extracted from earhole biopsies. *Cpa3*$^{cre/wt}$ mice were genotyped by PCR using a combination of three oligonucleotides (common 5': GGA CTG TTC ATC CCC AGG AAC C; 3'-WT: CTG GCG TGC TTT TCA TTC TGG;133'-KI: GTC CGG ACA CGC TGA ACT TG), yielding 320 bp (Cpa3^{+}) and 450 bp (Cpa3^{cre}) products for heterozygous *Cpa3*^{cre} mice. Cycling conditions were as followed: 15 min polymerase activation at 95°C, 40 cycles at 95°C for 30 sec, 57°C for 30 sec and 72°C for 40 sec.
