# Supplementary material for: Limited role of mast cells during infection with the parasitic nematode Litomosoides sigmodontis
Source: PLoS Negl Trop Dis. 2020 Jul 31;14(7):e0008534. doi: 10.1371/journal.pntd.0008534 (PMC7423137; doi:10.1371/journal.pntd.0008534)
Supplement: S2 Fig — Representative dot blots showing the gating strategy. Thoracic cavity cells were isolated from noninfected mice (A) and day 30 L. sigmodontis infected mice (B). Mast cells were defined as lineage (CD4, CD8 and CD19)- CD11b- leukocytes that are c-Kit (CD117)+ and IgE+. 1 x 10^6^ cells were gated for the analysis. (PDF) [file pntd.0008534.s002.pdf]

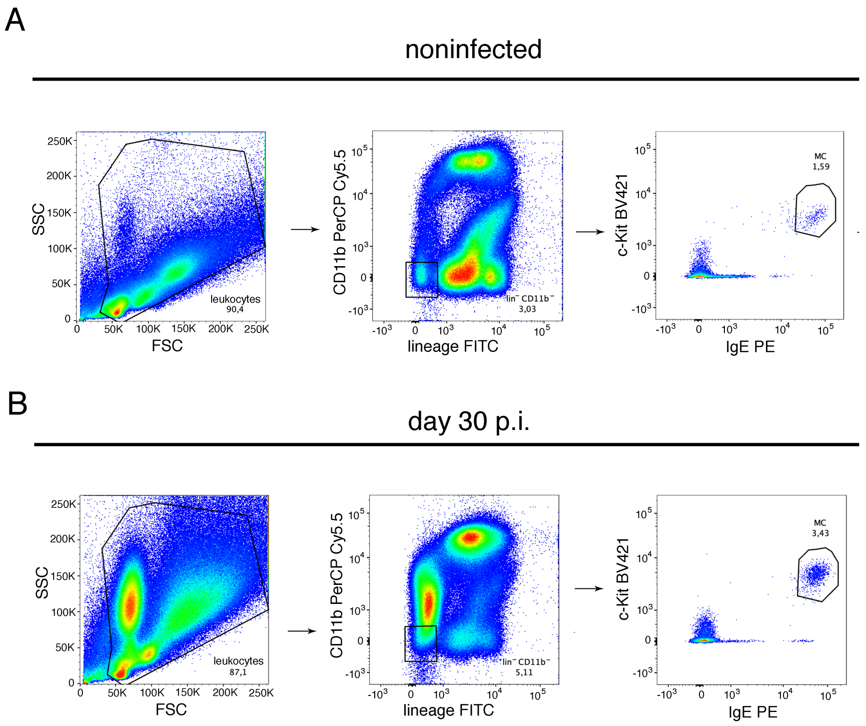

S2 Fig: Gating strategy.

Representative dot blots showing the gating strategy. Thoracic cavity cells were isolated from noninfected mice (A) and day 30 *L. sigmodontis* infected mice (B). Mast cells were defined as lineage (CD4, CD8 and CD19)$^{-}$ CD11b^{-} leukocytes that are c-Kit (CD117)$^{+}$ and IgE^{+}. 1 x 10^{6} cells were gated for the analysis.
